# A Hierarchical Grid Solver for Simulation of Flows of Complex Fluids

**DOI:** 10.3390/polym13183168

**Published:** 2021-09-18

**Authors:** Antonio Castelo, Alexandre M. Afonso, Wesley De Souza Bezerra

**Affiliations:** 1 Departamento de Matemática Aplicada e Estatística, Instituto de Ciências Matemáticas e de Computação, Universidade de São Paulo, Cx.P. 668, São Carlos 13560-970, SP, Brazil; 2 Centro de Estudos de Fenómenos de Transporte, Departamento de Engenharia Mecânica, Faculdade de Engenharia da Universidade do Porto, 4200-465 Porto, Portugal; aafonso@fe.up.pt; 3 Faculdade de Ciências Exatas e Tecnologia, Universidade Federal da Grande Dourados, Cx.P. 364, Dourados 79804-970, MS, Brazil; bezerra.ifsc@gmail.com

**Keywords:** finite difference methods, meshless interpolation, numerical solution, polymer flows, viscoelastic flows

## Abstract

Tree-based grids bring the advantage of using fast Cartesian discretizations, such as finite differences, and the flexibility and accuracy of local mesh refinement. The main challenge is how to adapt the discretization stencil near the interfaces between grid elements of different sizes, which is usually solved by local high-order geometrical interpolations. Most methods usually avoid this by limiting the mesh configuration (usually to graded quadtree/octree grids), reducing the number of cases to be treated locally. In this work, we employ a moving least squares meshless interpolation technique, allowing for more complex mesh configurations, still keeping the overall order of accuracy. This technique was implemented in the HiG-Flow code to simulate Newtonian, generalized Newtonian and viscoelastic fluids flows. Numerical tests and application to viscoelastic fluid flow simulations were performed to illustrate the flexibility and robustness of this new approach.

## 1. Introduction

Many researchers are constantly working on improving numerical solution techniques for partial differential equations that govern the flow of Newtonian and non-Newtonian fluids. One of the major problems faced is the part that generates the geometry of the problem to be simulated.

Cartesian hierarchical grids, or tree-based grids are the most common choices for discretizing the spatial domain. This choice allows the implementation of the finite difference method, while avoiding working with more complicated stencils, which occurs for example in curved meshes. Thus, it becomes easier to process the flow properties when a refinement of the mesh is desired in a determined region of the domain, since in a Cartesian grid, the flows are calculated in facets parallel to the Cartesian axes, favoring the implementation of the numerical method [1]. In the literature, quadtree and octree are 2D and 3D meshes, respectively, generated to perform these problems. One can say that a hierarchical grid is a generalization of quadtree and octree. In this sense, the choice of hierarchical grids is convenient to address the problem of flows in complex geometries [2,3,4,5,6].

Regarding the mesh refinement, one of the difficulties is to calculate the flow property value on these interfaces. High-order interpolations are commonly used. Several improvements of the interpolation techniques have been studied [7,8,9,10,11], in order to optimize the number of cells used in calculations, since this influences the computational time and storage over simulations.

In this way, the HiG-Fow system makes interpolations using the method of moving least squares, adapting the stencil according to the interface between the fine and coarse grids. Sousa et al. developed this methodology and compared it with non-graded methods by using the new system to simulate Newtonian flows [12].

Our interest is to use the HiG-Flow for the simulation of non-Newtonian flows. In this way, a code module for simulations of non-Newtonian flows was implemented, taking into account considerations shown in Section 3.

Depending on the temperature or mixture in liquid solvents, polymeric materials behave similar to viscoelastic fluids [13]. In this work we show that a new computer system is able to perform numerical simulations of viscoelastic fluid flows in two and three dimensions in channels with complex geometries. In one of the most common applications, polymers are used to construct electronic devices. Thus, the study of viscoelastic fluids is important due to applications in science and technology and the use of numerical simulators can be useful for support in important decisions in the engineering design of any device. In general, the behavior of viscoelastic fluid can be described using an appropriate constitutive model. So, in addition to using HiG-Flow in Newtonian flows, the system has implemented a module to solve the constitutive equations through the kernel-conformation technique. [14]. Different constitutive models are implemented, among them the Oldroyd-B [15] and Phan-Thien–Tanner (PTT) [16], which we used as reference in this work. In the last 20 years several works involving the solution of these constitutive models have been published. In 1999, Dou and Phan-Thien [17] used the finite volume method to solve the flow in a channel of an Oldroyd-B fluid past a circular cylinder. Then, Alves et al. [18] showed the effect of a high-resolution scheme MINMOD [19] on an upper-convected Maxwell fluid solution, improving accuracy and increasing the convergence rate of the finite volume method and then they proposed a new high resolution scheme [20]. Later the article was published [21] with benchmark solutions for the flow of Oldroyd-B and PTT fluids in planar contractions. In the year 2005 Chinyoka et al. [22] studied the deformation of a circular drop of an Oldroyd-B fluid by applying the volume-of-fluid method for two-dimensional interfaces. Later, Tomé et al. [23] applied the finite difference method to simulate free surface flow of PTT fluid in three dimensional geometry. Then, Mompean et al. [24] investigated fluid flows using the Upper-Convected Maxwell (UCM) constitutive equation and an explicit algebraic model to develop an approximation that could be applied to the extrudate-swell problem. In 2012, Tom é et al. [25] applied the log-conformation technique to study three-dimensional viscoelastic flows for jet buckling analysis and later Oishi et al. [26] and Paulo et al. [27] continued studies in this same way.

In 2019, Tomé et al. [28] presented a solution method for the Giesekus model flow and proposed a new analytical solution for this problem. In 2019, Bezerra et al. [29] used HiG-Flow to perform the solution of electro-osmotic flow of a viscoelastic fluid, where they proposed an approximation for the vortices simulation in a nozzle. Shojaei et al. [30] investigated a generalized finite difference method using the weighted moving least squares procedure, in the same way of our proposed numerical solution. Corresponding with one of the proposals of this work, [31] used stabilization techniques in 2D and 3D viscoelastic fluid flows. In 2020, Guan et al. proposed a improved finite difference method and they checked its convergence. Recently, [32] presented a implementation and computational verification of KBKZ integral constitutive equations in hierarchical grids. More recently, ref. [33] performed a generalized finite differences method for flows in a dam.

The finite difference method was used in the discretization of equations. The HiG-Flow system was also implemented taking into account advances in the MAC-Marker and Cell method [34], allowing the implementation of several solution methods for the different terms of the equation of motion as well as the constitutive model solution. Convective terms in equations can be solved by high-accuracy methods. Moreover, we can say that the main novelty for the simulation of viscoelastic fluids is the kernel-conformation technique. The technique is already known, however, the differential is the manner it was implemented in, which allows the user to choose a numerical stabilizer easily—one just needs to enter the desired mathematical function, the derivative of this function and its inverse function. More details can be found in the Governing Equations section. Here, numerical stabilizers were used for Oldroyd-B flow solution in a 2D cavity and for a PTT fluid in a complex 3D geometry.

In Section 2, we show the finite differences method of the approximation used. Then, the governing equations and the constitutive models are presented in Section 3, as well as the explanation of the kernel-conformation technique. In Section 4, we present the validation tests for a PTT fluid flow in a pipe and to an Oldroyd-B fluid flow in a 2D-lid-driven cavity. Finally, we performed simulations of a PTT flow in a complex 3D geometry and the results are shown in Section 5.

## 2. Finite Difference Approximation in Tree-Based Grids

**Figure 1:**
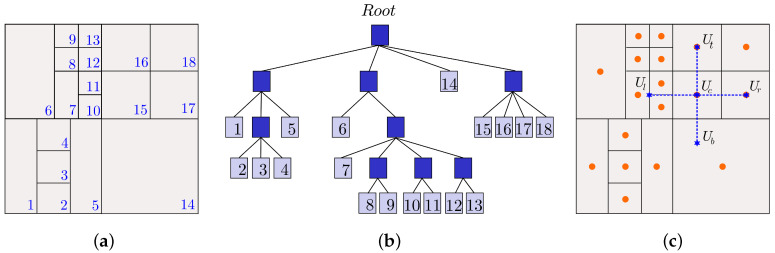
(**a**) Example of hierarchical grid. (**b**) Tree data structure. (**c**) Finite difference method.

In the HiG-Flow code, the equations are solved using finite difference approach in hierarchical meshes. Figure 1a shows a representative type of mesh and the dependency structure (tree data structure) is presented in Figure 1b. In this approach, cells can be partitioned into different geometrical shapes. Such generalization leads to the difficult task of finding an accurate approximation to the different differential and integral operators.

Looking at Figure 1c, a second-order approximation to $\frac{\partial^{2}U_{c}}{\partial y^{2}}$ can be given by (we assume the *y* axis is in the direction bottom → top):

(1) $$\frac{\partial^{2}U_{c}}{\partial y^{2}}\approx \frac{1}{\delta y}\left(U_{t}-2U_{c}+U_{b}\right).$$

Note that $U_{b}$ is not known and must be obtained by interpolation (the same applies to $U_{r}$) using the following formula:

(2) $$U_{b}=\sum_{k=1}^{V_{b}}w_{k}^{b}\;U_{k},$$

where $V_{b}$ is the number of neighbor cells, which depends on the imposed accuracy of the method.

The weights $w_{k}^{b}=w_{k}^{b}\left(x\right)$ are obtained through the moving least squares (MLS) method. In a set of *n* smooth interpolating functions that are linearly independent $\Phi_{i}:R^{d}\to R$ (d=2,3), we want to obtain the interpolated value *u* such that $U_{b}=U\left(x\right)=\sum_{k=1}^{n}c_{i}\Phi_{i}\left(x\right)=c^{t}\Phi$.

Given *m* points $x_{1},x_{2},\ldots ,x_{m}\in R^{d}$ with m>n and *m* values $u_{1}=u\left(x_{1}\right),u_{2}=u\left(x_{2}\right),\ldots ,u_{m}=u\left(x_{m}\right)$, to interpolate *u* in x using MLS consists in minimizing the error E(c)

(3) $$E\left(c\right)={∥U-u∥}_{2}^{2}=\sum_{i=1}^{m}{\left(U\left(x_{i}\right)-u_{i}\right)}^{2}\frac{1}{∥x-x_{i}{∥}_{2}}.$$

Or,

(4) $$E\left(c\right)={∥WPc-Wu∥}_{2}^{2},$$

where $W=W\left(x\right)=\left\{\delta_{ij}\sqrt{\frac{1}{∥x-x_{i}{∥}_{2}}}\right\}\in R^{m\times m}$, $P=\left\{\Phi_{i}\left(x_{j}\right)\right\}\in R^{m\times n}$ and $u=(u_{1},u_{2},\ldots ,u_{m})$.

The solution is given by

(5) $$c\left(x\right)={\left(WP\right)}^{†}Wu$$

where ${\left(\cdot \right)}^{†}$ is the Moore–Penrose pseudo-inverse.

Decomposing (WP) into QR we have that

(6) $$WP=Q\left[\begin{array}{c} R\\ 0 \end{array}\right]=\left[Q_{\|}\;\;Q_{⊥}\right]\left[\begin{array}{c} R\\ 0 \end{array}\right]\;,$$

where $Q\in R^{m\times m}$, $Q_{\|}\in R^{m\times n}$, $Q_{⊥}\in R^{m\times m-n}$, and $R\in R^{n\times n}$. This decomposition is then used to finally compute

(7) $$c\left(x\right)=R^{-1}Q_{\|}^{t}Wu\;.$$

Then

(8) $$U\left(x\right)=c^{t}\Phi =u^{t}\underset{w}{\underset{︸}{WQ_{\|}R^{-t}\Phi }}=\sum_{k=1}^{m}w_{k}u_{k}\;,$$

that is $w=w\left(x\right)=WQ_{\|}R^{-t}\Phi .$

The procedure to calculate w(x) must be performed for each approximation U(x). This is performed only once since we are using a static mesh.

## 3. Governing Equations

The flow is assumed to be isothermal, laminar and the fluids incompressible. The governing equations are those expressing conservation of mass

(9) ∇·u=0,

and conservation of momentum

(10) $$\frac{\partial u}{\partial t}+u\cdot \nabla u=-\nabla p+\frac{1}{Re}\nabla^{2}u+\nabla \cdot S+\frac{1}{Fr^{2}}g+F,$$

(11) $$T=\frac{2(1-\beta )}{Re}D+S,$$

where u is the velocity field, *t* is time, *p* is the pressure, Re is the Reynolds number, Fr is the Froude number, g is the gravity force and F is the surface tension force and source force. The symbol $D=\frac{1}{2}\left(\nabla u+{\left(\nabla u\right)}^{T}\right)$ is the rate of deformation tensor, T is the elastic stress. The amount of Newtonian solvent is controlled by the dimensionless solvent viscosity coefficient, $\beta =\frac{\mu_{S}}{\mu_{0}}$, where $\mu_{0}=\mu_{S}+\mu_{P}$ denotes the total shear viscosity. Several polymeric constitutive equations are implemented in the current version of the solver: the upper-convected Maxwell model, the Oldroyd-B model, the linear form of the Phan-Thien/Tanner (LPTT) model [35] and the Giesekus model [36]. For an isothermal flow, these five rheological equations of state can be written in a compact form as:

(12) $$\frac{\partial T}{\partial t}+\left(u\cdot \nabla \right)T-\left[{\left(\nabla u\right)}^{T}\cdot T+T\cdot \nabla u\right]=\frac{1}{De}M\left(T\right).$$

where $M\left(T\right)$ is defined by the viscoelastic model

(13) $$M\left(T\right)=\left\{\begin{array}{cc} \frac{2(1-\beta )}{Re}D-T & \mathrm{Oldroyd}-B,\\ \frac{2(1-\beta )}{Re}D-T-\frac{\alpha \;Re\;De}{1-\beta }T\cdot T & \mathrm{Giesekus},\\ \frac{2(1-\beta )}{Re}D-\left(1+\frac{\epsilon \;Re\;De}{1-\beta }\mathrm{tr}\left(T\right)\right)T-\xi \;De\left(T\cdot D+D\cdot T\right) & \mathrm{LPTT}, \end{array}\right.$$

where De is the Deborah number. The stress coefficient function of the LPTT model depends on the trace of T, $\mathrm{tr}\left(T\right)$ and introduces the dimensionless parameter ϵ, which is closely related to the steady-state elongational viscosity in extensional flows. The slip parameter, ξ, takes into account the non-affine motion between the polymer molecules and the continuum. The polymer strands embedded in the medium may slip with respect to the deformation of the macroscopic medium, thus each strand may transmit only a fraction of its tension to the surrounding continuum. When ξ=0 there is no slip and the motion becomes affine. Parameter ξ is responsible for a non-zero second normal-stress difference in shear, leading to secondary flows in ducts having non-circular cross-sections, which is superimposed on the streamwise flow. In the non-linear term of the Giesekus model, α represents a dimensionless “mobility factor”.

An alternative form to describe viscoelastic models is by using the conformation tensor, A. This tensor is Symmetric and Positive Definite (SPD), which is an important mathematical property for the construction of matrix transformations and/or decompositions. In general, the equation for A can be written as

(14) $$\frac{\partial A}{\partial t}+\left(u\cdot \nabla \right)A-\left[A\nabla u+\nabla u^{T}A\right]=\frac{1}{De}M\left(A\right),$$

where M(A) is a function that depends on the specific constitutive model. The relation between stress tensor T and A is given by

(15) $$T=\frac{1-\beta }{Re\;De}\left(A-I\right),$$

that can rewritten as a relation between the tensor S and A given by

(16) $$S=\frac{1-\beta }{Re\;De}\left(A-I-2De\;D\right).$$

A problem that challenges many researchers in computational rheology is solving Equation (12) or Equation (14) for high values of the Deborah number, $De=\lambda /t_{c}$, where $t_{c}$ is a characteristic time of the flow. This problem occurs because all numerical methods are unstable for certain critical values of De. In order to overcome such failure, Fattal and Kupferman [37] proposed a reformulation of the differential constitutive equations into a equation for the matrix logarithm of the conformation tensor. Extending the ideas proposed by [37,38], ref. [14] presented a generic kernel-conformation tensor transformation that allows us to apply different kernel functions to the matrix transformation.

The reformulation of the tensor conformation was possible by the decomposition of the velocity gradient proposal by [37,38]

(17) $$\nabla u^{T}=\Omega +B+NA^{-1},$$

where Ω and N are anti-symmetric tensors, B is symmetric and commutes with A. Thus, the constitutive equation based on the conformation tensor can be rewritten using the decomposition (17) as

(18) $$\frac{\partial A}{\partial t}+\left(u\cdot \nabla \right)A-\left(\Omega A-A\Omega \right)-2BA=\frac{1}{De}M\left(A\right),$$

where M(A) is defined according to the viscoelastic model,

(19) $$M\left(A\right)=\left\{\begin{array}{cc} I-A & \mathrm{Oldroyd}-B,\\ I-A-\alpha \left(A-I\right)\cdot \left(A-I\right) & \mathrm{Giesekus},\\ \left(1+\frac{\epsilon \;Re\;De}{1-\beta }\mathrm{tr}\left(S\right)\right)\left(I-A\right)-2\xi \;De\left(B-BA\right) & \mathrm{PTT}. \end{array}\right.$$

Fattal and Kupferman showed that the matrix logarithm of the conformation tensor is a linear transformation of A and derived a constitutive equation from the Equation (18) in the function of the matrix logarithm. Afonso et al. proposed a generic *kernel-conformation* tensor transformation for a large class of differential constitutive models, in which the evolution equation for k(A), can be expressed in its tensorial formulations as

(20) $$\frac{Dk(A)}{Dt}=\Omega k\left(A\right)-k\left(A\right)\Omega +2B+\frac{1}{De}M$$

where B and M are symmetric tensors constructed by the orthogonalization of the diagonal tensors $D_{B}$ and $D_{M}$, respectively. These tensors can be constructed as

(21) $$\begin{array}{ccc} B & =OD_{B}O^{T}= & O\tilde{B}\Lambda JO^{T}\\ M & =OD_{M}O^{T}= & OM\left(\Lambda \right)JO^{T}. \end{array}$$

In Equations (21), J is the gradient matrix, a diagonal matrix of the form,

(22) $$J=\mathrm{diag}\left(\frac{\partial k\left(\lambda_{1}\right)}{\partial \lambda_{1}};\frac{\partial k\left(\lambda_{2}\right)}{\partial \lambda_{2}};\frac{\partial k\left(\lambda_{3}\right)}{\partial \lambda_{3}}\right).$$

## 4. Verification Tests

In this section, we address two test problems in terms of checking the HiG-Flow code for simulations of viscoelastic flows. One of the problems is the flow of a Phan-Thien–Tanner model fluid in a circular cross-section channel. The other test problem concerns the constitutive model of Oldroyd-B. The geometry used for this test was a driven cavity in two dimensions.

### 4.1. Phan-Thien–Tanner Model Fluid Flow in a Pipe

We consider a flow into a circular cylinder of radius *R*, where there exists only the axial velocity component *u*, which depends on the radial coordinate *r*. In addition, we consider that the fluid obeys the PTT fluid model [16] and the flow occurs in the *x* direction, the same as the cylinder axis. Here, we consider the known solutions available of the literature to the flow properties, namely velocity *u*, shear stress $T_{rx}$ and normal stress $T_{xx}$. More detailed treatment about the analytical solution to this problem in a steady state, as well as the results verified here, can be found in [39,40,41]. Essentially, to obtain the viscoelastic component $T_{rx}$, it is necessary to solve a cubic equation $T_{rx}^{3}+3AT_{rx}-2B=0$, whose its solution is given by

(23) $$T_{rx}={\left[B+{\left(A^{3}+B^{2}\right)}^{1/2}\right]}^{1/3}+{\left[B-{\left(A^{3}+B^{2}\right)}^{1/2}\right]}^{1/3},$$

where *A* and *B* depends on the set of know parameters of flow:

(24) $$\begin{array}{cc} A & =\frac{\eta_{p}^{2}}{6\varepsilon \lambda^{2}\beta }, \end{array}$$

(25) $$\begin{array}{cc} B & =-\frac{\eta_{p}^{3}u_{N}}{\varepsilon \lambda^{2}R^{2}\beta }r. \end{array}$$

In Equations (24) and (25), $\eta_{p}$ is the polymer viscosity, ε is the PTT parameter, λ is the relaxation time and *R* is the cylinder radius. The amount of solvent contribution is given by $\beta =\eta_{s}/\eta_{0}$, the reference velocity is $u_{N}$ and *r* is the radial coordinate. After obtaining $T_{rx}$, one can calculate the normal stress $T_{xx}$ and also integrate the equation of motion to determine the velocity field. The corresponding expressions are given by:

(26) $$\begin{array}{cc} T_{xx} & =\frac{2\lambda }{\eta_{p}}T_{rx}^{2}, \end{array}$$

(27) $$\begin{array}{cc} u(r) & =\frac{2u_{N}}{\beta }\left[1-{\left(\frac{r}{R}\right)}^{2}\right]+f\left(A,B\right), \end{array}$$

where f(A,B) is a function that depends on the parameters *A* and *B* given in (24) and (25), respectively. These simulation parameters can be adjusted when the polymer viscosity is fixed, then by varying β it is possible to control the amount of the solvent contribution. In addition, just as β, ε and De are input viscoelastic parameters, λ is adjusted by the Deborah number. To verify that the results are in agreement with [41], we set ε=0.25 and De=6.3, which corresponds to the reference $De_{N}=1.0$. Non-slip boundary conditions were used for the velocity in the cylinder wall. At the channel inlet, we imposed a parabolic velocity profile and at the outlet, the homogeneous Neummann boundary condition, that is, spatial variations in velocity are not allowed at the outflow. For pressure, a zero gradient was imposed on the wall and at the channel inlet while the outflow was fixed at a constant value. The initial conditions for the bulk domain is zero velocity.

**Figure 2:**
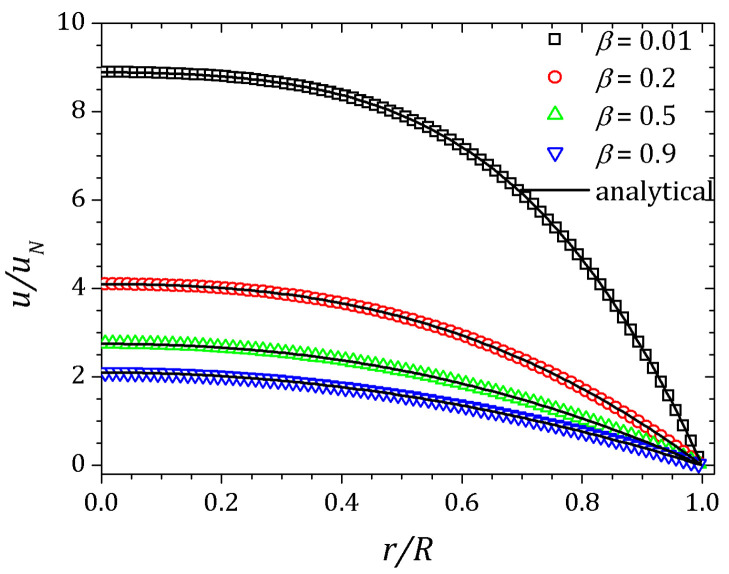
Velocity field for a PTT flow in a pipe.

**Figure 3:**
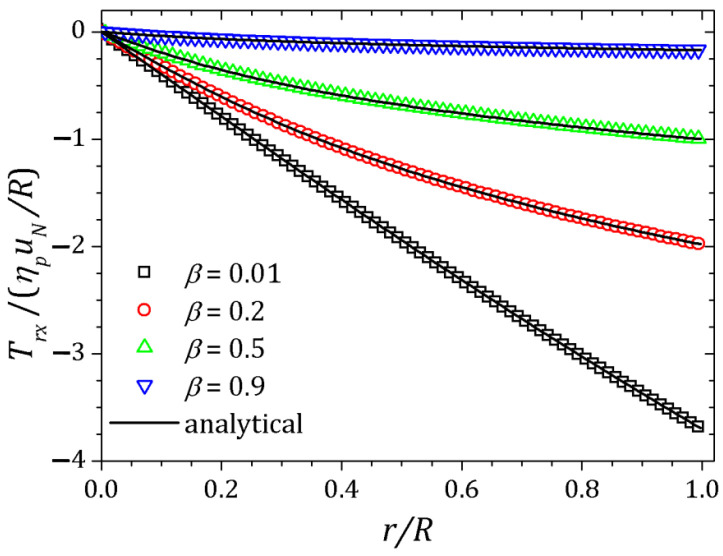
Shear stress $T_{rx}$ for a PTT flow in a pipe.

**Figure 4:**
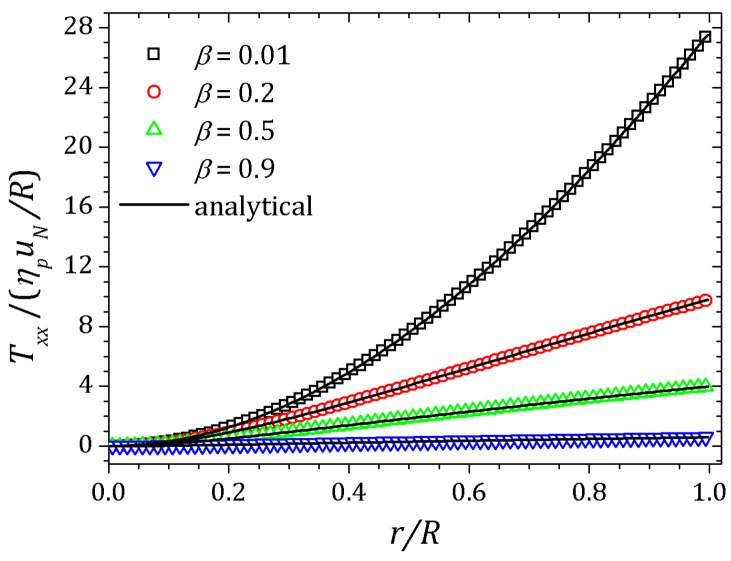
Normal stress for a PTT flow in a pipe.

Figure 2, Figure 3 and Figure 4 show the velocity field, shear stress and normal stress, respectively, as a function of the amount of solvent, which is controlled by β parameter. When β≈1, the polymer concentration is approximately zero and the fluid has Newtonian behavior. On the other hand, if β≈0, the behavior of the PTT fluid resembles that of the Oldroyd-B model. The curves represented by down triangles corresponds to β=0.9, up triangles to β=0.5, circles to β=0.2 and squares to β=0.01. All these results are obtained by HiG-Flow simulation. They are in perfect agreement with the analytical curves represented by solid lines in Figure 2, Figure 3 and Figure 4, which corresponds to the solutions given by Equations (23), (27) and (26) for *u*, $T_{rx}$ and $T_{xx}$, respectively.

### 4.2. 2D-Driven Cavity with Oldroyd-B Flow

Flows in rectangular cavities have been studied since 1967 when the article [42] was published. In the 21st century, several studies of this type for viscoelastic fluids have been published [38,43,44,45,46,47]. The problem studied here has no analytical solution; however, there are results obtained by the cited authors that can be used for comparison. The data used for comparison in this study were provided by Palhares Junior et al. [47].

**Figure 5:**
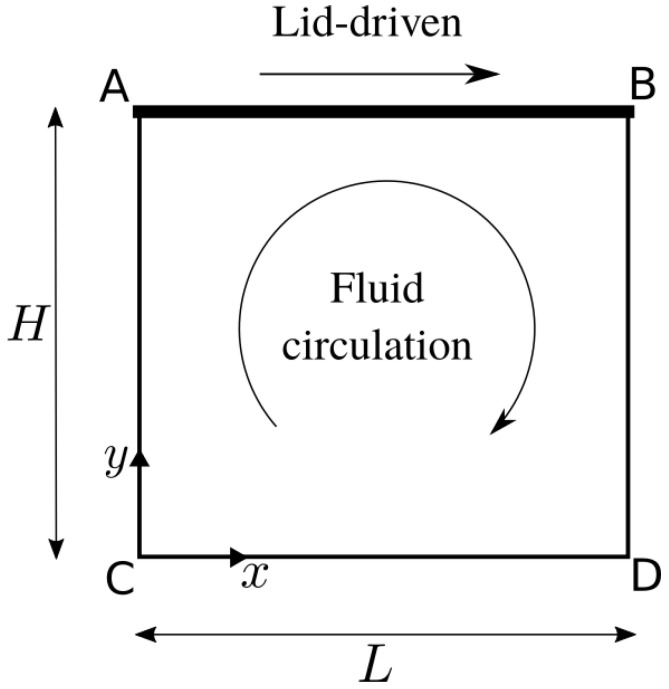
Illustration of lid-driven cavity. The parabolic velocity profile is imposed on the top. The aspect ratio is defined as Λ≡H/L.

Figure 5 shows the schematic of lid-driven cavity. A parabolic profile velocity is imposed on the top. The aspect ratio is defined as Λ≡H/L. Some concerned works are listed in Table 1.

The use of stabilization methods within the HiG-Flow system can be considered simple from the coder point of view because the code has been implemented in such a way that one can make a choice directly in the main simulation file. In this sense, the kernel conformation tensor is used to perform this operation, as previously described in the Section 3. Generically, the user simply writes the kernel k, the kernel derivative dk/dx for the Jacobian transformation calculation and the kernel inverse $k^{-1}$ correspondents. For the square root stabilizer used in these simulations, one can write Equations (28)–(30), respectively, as follows:

(28) $$\begin{array}{cc} k_{ij} & =\sqrt{S_{ij}}, \end{array}$$

(29) $$\begin{array}{cc} \frac{dk_{ij}}{dx} & =\frac{1}{2\sqrt{S_{ij}}}, \end{array}$$

(30) $$\begin{array}{cc} k_{ij}^{-1} & =S_{ij}^{2}, \end{array}$$

For the simulations, the Reynolds number was fixed as Re=0.01. The proportion of solvent in Oldroyd-B fluid was also fixed as β=0.5. Simulations were performed for two different Deborah numbers, De=1.0 and De=2.0. On the top lid-driven section of the cavity, we imposed a parabolic velocity profile given by

(31) $$u\left(x,t\right)=8\left[1+\tanh(8t-4)\right]x^{2}{\left(1-x^{2}\right)}^{2}.$$

**Figure 6:**
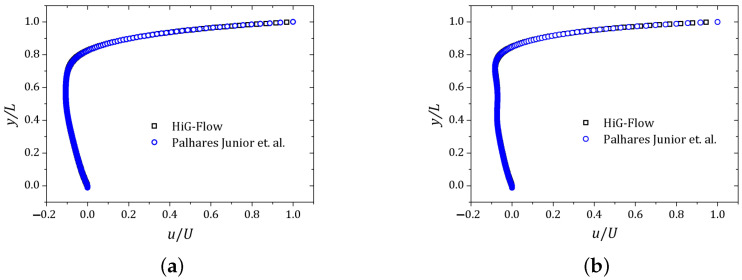
Velocity field *u* obtained along the vertical line x=0.5: (**a**) De=1.0; (**b**) De=2.0.

**Figure 7:**
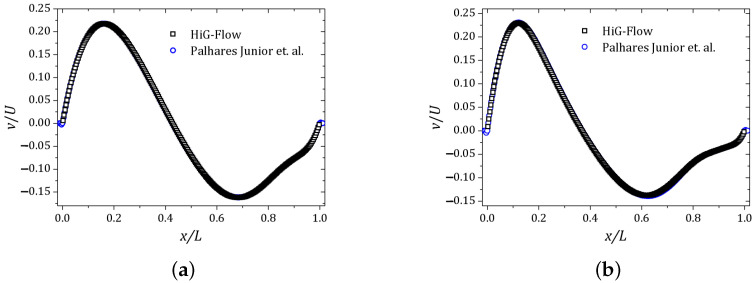
Velocity field *v* obtained along the horizontal line y=0.75: (**a**) De=1.0; (**b**) De=2.0.

**Figure 8:**
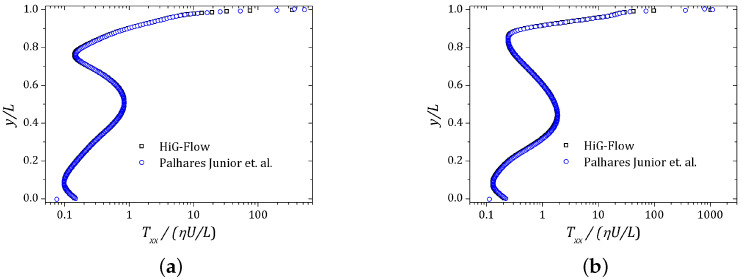
Normal stress $T_{xx}$ obtained along the vertical line x=0.5: (**a**) De=1.0; (**b**) De=2.0.

The other cavity walls are stationary and the non-slip condition is imposed over all of them. A regular mesh of 256 × 256 cells was used. The velocity component *u* and the normal stress $T_{xx}$ were plotted along the vertical line x=0.5 while the velocity component *v* was obtained on the horizontal line y=0.75. The (x,y) coordinates are scaled by the cavity side size L=1 unit of length. The results are shown in Figure 6, Figure 7 and Figure 8. The curves are represented by squares and circles corresponding to the HiG-Flow and Palhares Junior et al. results, respectively. The graphs indicate that the results are in good agreement.

## 5. Simulation in Complex 3D Array of Channels

In this section, we present the results obtained with the HiG-Flow system for flows in complex domains. We simulated an incompressible viscoelastic fluid flow in a complex array of microchannels, introducing some level of geometric complexity in the three-dimensional flow domain.

**Figure 9:**
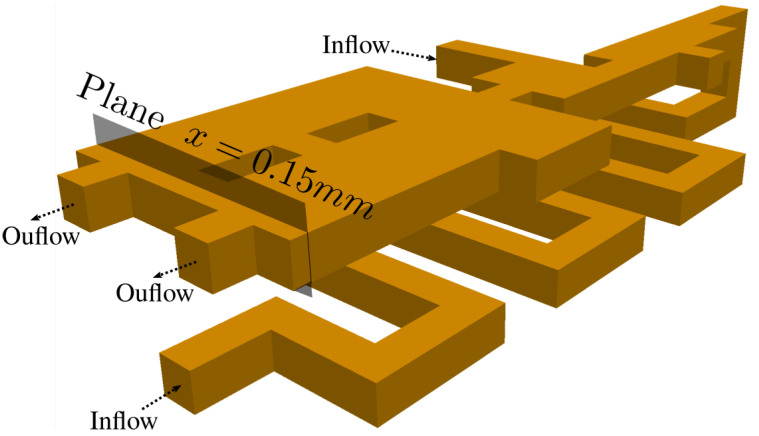
Geometry for the complex 3D array of channels.

The geometry, as well as boundary conditions, can be seen in Figure 9. The total width, length and height are set to be W=0.8 mm, L=2.4 mm and H=0.4 mm, respectively. The inlet is a channel of 0.1 mm × 0.1 mm, where polymer at temperature is injected with a constant velocity of $U_{\mathrm{in}}=0.1$ mm/s. Scaling this geometry by ℓ=0.1 mm, and using $\nu \approx 10^{-4}$ m${}^{2}$/s as the kinematic viscosity of polymer at room temperature, we end up with a Reynolds number of $Re=\ell \;U_{\mathrm{in}}/\nu =1.0$. In this test, we used the PTT model with β=0.5, ε=0.25 and ξ=0.0 for several values of De=[0−500] as viscoelastic parameters.

**Figure 10:**
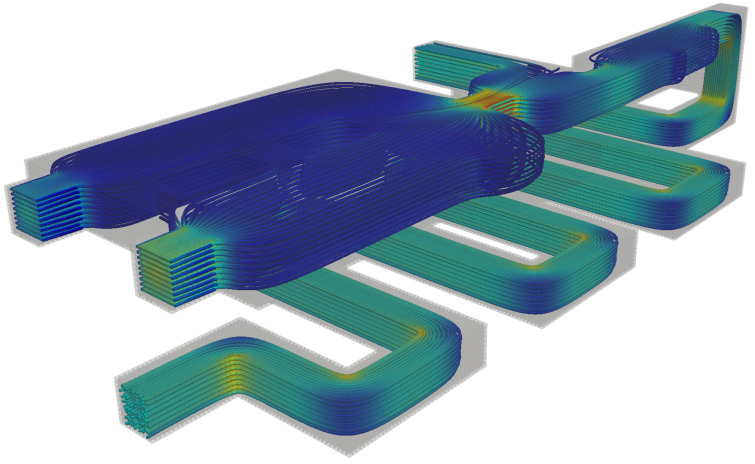
Streamlines for the complex 3D array of channels. The color scale varies from smallest (blue) to largest (red) velocity magnitude.

**Figure 11:**
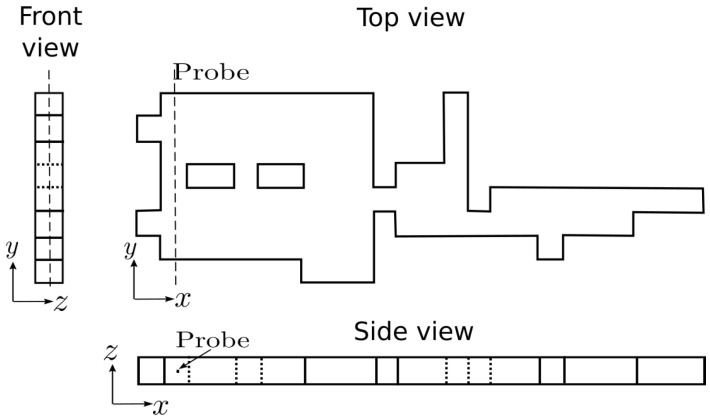
Probe views.

Streamlines for the flow of a Newtonian fluid can be observed in Figure 10. The result is qualitative, but demonstrates the robustness and applicability of this newly developed methodology. Several simulations using viscoelastic fluids for De=[0−500] were performed on the 3D complex geometry. We analyzed the complex fluid flow by observing the profiles of the polymeric stresses along the probe line near the 3D channel exits, as shown in Figure 11. The probe is aligned on the *y* direction at half channel height (along the *z* direction), orthogonal to the main flow direction near the channel exits.

**Figure 12:**
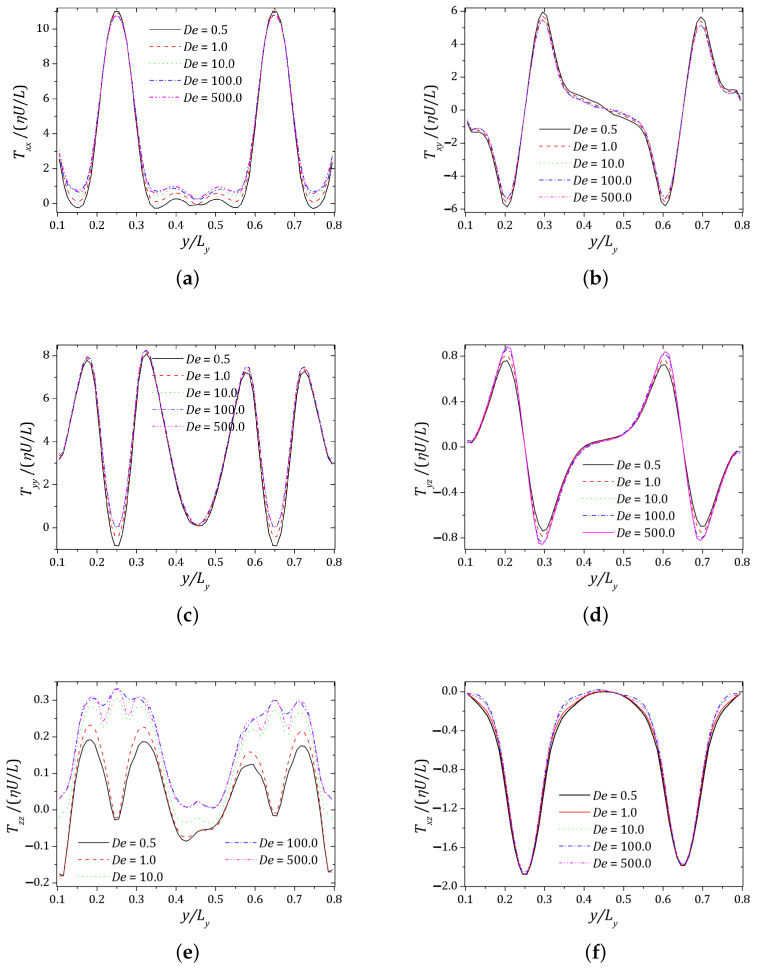
Tensor components: (**a**) $T_{xx}$; (**b**) $T_{xy}$; (**c**) $T_{yy}$; (**d**) $T_{yz}$; (**e**) $T_{zz}$; (**f**) $T_{xz}$.

The increasing values of elasticity, reflected on the value of Deborah number represented in Figure 12, affects the six components of the non-dimensional extra stress tensor along the probe line, with higher impact for the normal components, as the $T_{zz}$ profiles. Nevertheless, given that no geometrical singularity is presented along the probe line, the maximum value for all extra stress components is not significant and slightly affected by the increase in elasticity.

We used a computer with a 3.1 GHz Intel Core i7 Quad-Core Processor and 16 GB 2133 MHz LPDDR3 memory. The HiG-Flow software was used with four cores for all the calculation, and each simulation took 14 h of processing.

## 6. Conclusions

Tree-based grids bring the advantage of using fast Cartesian discretizations, such as finite differences, and the flexibility and accuracy of local mesh refinement. Most methods usually avoid this by limiting the mesh configuration (usually to graded quadtree/octree grids), reducing the number of cases to be treated locally. In this work, we employ a moving least squares meshless interpolation technique, allowing for more complex mesh configurations, while still keeping the overall order of accuracy. This technique was implemented in the HiG-Flow code to simulate Newtonian, generalized Newtonian and viscoelastic fluids flows. The code verification and testing was performed using numerical stabilizers for the Oldroyd-B flow solution in a 2D cavity and for a PTT fluid in a complex 3D geometry.

## Figures and Tables

**Table 1:** Previous and current numerical studies concerned with lid-driven cavity flow of constant viscosity viscoelastic fluids.

Reference	Aspect Ratios	Constitutive Equation	*De*	Regularization	Notes
Grillet et al. [48]	0.5, 1.0, 3.0	FENE-CR, $L^{2}=$ 25,100,400	≤0.24	Leakage at corners A and B	FE
Fattal and Kupferman [38]	1.0	Oldroyd-B, β=0.5	1.0,2.0,3.0,5.0	$u\left(x\right)=16Ux^{2}{\left(1-x\right)}^{2}$	FD, Log conformation technique
Pan et al. [43]	1.0	Oldroyd-B, β=0.5	0.5,1.0	$u\left(x\right)=16Ux^{2}{\left(1-x\right)}^{2}$	FE, Log conformation technique
Yapici et al. [44]	1.0	Oldroyd-B, β=0.3	≤1.0	No	FV, First-order upwind
Habla et al. [46]	1.0	Oldroyd-B, β=0.5	0 to 2	$u\left(x,z\right)=128\left[1+\tanh8(t-1/2)\right]$ $x^{2}{\left(1-x\right)}^{2}z^{2}{\left(1-z\right)}^{2}$	FV, 3D, Log conformation technique, CUBISTA
Comminal et al. [49]	1.0	Oldroyd-B, β=0.5	0.25 to 10	$u\left(x\right)=16Ux^{2}{\left(1-x\right)}^{2}$	FD/FV, Log-conformation, stream function
Martins et al. [50]	1.0	Oldroyd-B, β=0.5	0.5,1.0,2.0	$u\left(x\right)=16Ux^{2}{\left(1-x\right)}^{2}$	FD, Kernel-conformation technique
Dalal et al. [51]	1.0	Oldroyd-B, β=0.5	1.0	$u\left(x\right)=16Ux^{2}{\left(1-x\right)}^{2}$	FD, Symmetric square root
Palhares Junior et al. [47]	1.0	Oldroyd-B, β=0.5	1.0,2.0	$u\left(x,t\right)=8\left[1+\tanh(8t-4)\right]x^{2}$ ${\left(1-x^{2}\right)}^{2}$	FD, Symmetric square root
Current work	1.0	Oldroyd-B, β=0.5	1.0,2.0	$u\left(x,t\right)=8\left[1+\tanh(8t-4)\right]x^{2}$ ${\left(1-x^{2}\right)}^{2}$	FD, Kernel-conformation technique
